# A simulation framework for evaluating electronic order workflows in integrated health records

**DOI:** 10.1038/s44401-026-00067-2

**Published:** 2026-02-02

**Authors:** Yang Chen, Haoran Niu, Olufemi A. Omitaomu, Soumendra Bhanja, Angela Laurio, Amber Trickey, Vijayalakshmi Sampath, Jonathan R. Nebeker

**Affiliations:** 1 Computational Sciences and Engineering Division, Oak Ridge National Laboratory, Oak Ridge, TN USA; 2 Digital Health Office, US Department of Veterans Affairs, Washington, DC USA; 3 Salt Lake City Geriatrics Research and Education Clinical Center, US Department of Veterans Affairs, Salt Lake City, UT USA; 4 Department of Medicine, University of Utah, Salt Lake City, UT USA

**Keywords:** Engineering, Health care, Mathematics and computing

## Abstract

Electronic health record (EHR) systems are critical to modern healthcare delivery, yet the dynamic workflows that govern electronic order processing remain underexplored. Inefficiencies in these digital pathways can cause delays in care, repetitive workloads, and even patient harm. This study presents a discrete-event simulation framework used to reconstruct and evaluate EHR-based order workflows in a large integrated healthcare system. Using real-world data extracted from the Veterans Health Administration’s Corporate Data Warehouse, the authors mapped order events to standardized state transitions and modeled their progression across different facilities of varying complexity levels. After being calibrated with empirical distributions of transition times and validated against observed time-in-system metrics, the simulation demonstrates close alignment with historical performance. Scenario analyses reveal that resource capacity constraints significantly amplify the impact of electronic order surges, which are reflected in the disproportionate growth in backlogs and processing delays. Adjustments in transition probabilities further increased recirculation and extended workflow paths. Network-based analysis identified *Reserved*, *InProgress*, and *Completed* as structurally critical states that function as hubs within the process network but the transitions in-between also act as major bottlenecks. These results showcased the effectiveness of simulation-based approaches in monitoring EHR order processing performance and evaluating consequences of workflow changes on healthcare network resources planning. The proposed simulation framework provides a scalable data-driven tool to support operational decision-making and improve the efficiency of electronic order management in complex healthcare environments.

## Introduction

The electronic health record (EHR) system is a cornerstone of Health Information Technology (HIT). EHRs enable digital management of patient health information and support the coordination of care across complex organizations. In the United States, the Veterans Health Administration (VHA) operates the country’s largest integrated healthcare system, serving over 9 million enrolled veterans through 1380 facilities, including 170 medical centers and 1193 outpatient sites across the country ^1^. Since the 1990s, the Veterans Health Information Systems and Technology Architecture (VistA)^2^ has provided the backbone of the VHA’s HIT (e.g., the Corporate Data Warehouse [CDW]^3^) to facilitate data exchange and care coordination. Recognizing persistent challenges related to system fragmentation and interoperability, the US Department of Veterans Affairs (VA) launched a multi-billion-dollar modernization initiative in 2017 to transition from VistA to Federal EHR ^4,5^. This effort aims to streamline order management workflows among healthcare providers by unifying EHR systems between the VHA and the US Department of Defense to provide a single, longitudinal health record that contains a given veteran’s full medical history, which will help reduce prescription errors, prevent redundant testing, and ultimately improve the quality of veteran healthcare. Although the anticipated benefits of a consolidated EHR system are substantial, the transition has introduced complexities and challenges in areas such as data migration, clinician training ^6^, workflow adaptation, and system usability ^7,8^. These challenges underscore the need for tools to monitor operational performance and evaluate the impact of change to maximize the benefits of this modernization effort.

The dynamic and complex nature of healthcare systems, especially within large integrated networks such as those of the VHA, presents significant challenges in predicting the downstream effects of operational changes. Traditional evaluation methods (e.g., retrospective data analysis, observational studies) often fall short in capturing the intricate interdependencies among clinical process, resource allocation, and patient care outcomes. For instance, process mining research in health care domain has been reviewed ^9^ from three novel dimensions of process mining project stages, involvement of domain expertise and key performance indicators. It is found that process redesign is rarely part of a process mining project, domain experts are mostly asked for validating insights, and less than half of the published papers consider one or more specific performance indicators to direct the analysis. To address these challenges, simulation-based approaches such as discrete-event simulation (DES), agent-based modeling (ABM), and system dynamics (SD) have been widely employed in healthcare research because they can provide valuable insights into operational efficiency optimization (e.g., patient wait time reduction) and care delivery improvement^10^. Among these simulation approaches, the number of publications that describe the use of DES methods has increased significantly since 2004^11^. For example, Kalton et al. developed a multi-agent simulation to evaluate care coordination for patients with serious mental illnesses by simulating interactions between patients, healthcare providers, social services, and the criminal justice system. Using real-world data and expert input, the model demonstrates how enhanced coordination reduces crises and improves patient outcomes^12^. Vahdat et al. examined the effects of increased provider-patient encounter times caused by EHR implementation in an outpatient dermatology clinic. The results show that even small increases in in-room documentation time (1–10 minutes) led to significant delays, with wait times rising by 22%–373% and length of stay increasing by 12%–193%. To offset these delays, clinics would need to reduce patient volume by up to 50% to maintain performance^13^. Simwita et al. applied DES to evaluate resource flexibility in an orthopedic care process at Bugando Medical Centre in Tanzania^14^ and demonstrated that reallocating mid-level healthcare workers for routine tasks to optimize the use of specialized surgeons can reduce patient waiting times by up to 72.7% and increase throughput by 94%. Despite DES being increasingly applied to operational healthcare issues, fewer than 10% of simulation studies progress beyond the modeling stage to real-world implementation^15^. In addition to standalone simulation methods, hybrid simulation approaches have emerged to capture multilevel healthcare processes. Djanatliev et al. introduced a multi-paradigm simulation approach that forms an SD-ABM-DES framework to support prospective health technology assessment^16^. After reviewing the hybrid simulation applications, Santos et al. reported the most popular combination is DES-SD, with AnyLogic as the predominant simulation tool^17^.

Although simulation-based research helps enable testing interventions and examine rare events that may be inaccessible in reality, it faces challenges in validity and applicability. However, integrating simulation with other methods can enhance its applicability and make it a promising approach for healthcare improvement research^18,19^. After reviewing 231 papers on DES modeling in healthcare^15^, around 66% of theoretical studies incorporate DES alongside other analytical techniques (e.g., optimization and Lean/Six Sigma). For example, DES is integrated with lean management and a business game to improve patient flow in an outpatient hematology-oncology clinic during a Kaizen event^20^. Kovalchuk et al. combined process mining, data mining, and DES^21^ to simulate patient flow in acute coronary syndrome care and show how empirical data can enhance simulation fidelity and predictive capacity. As another example, PartiSim is a multi-methodology framework that combines DES with a soft systems methodology to enhance stakeholder involvement throughout the study life cycle^22^. The framework features a comprehensive six-stage process that guides stakeholders from problem identification to solution implementation and has been successfully applied in two real-world studies. Kuruppu Appuhamilage et al. developed a dual-layer digital twin framework that combines DES and Azure cloud infrastructure^23^ to capture critical care workflows in real time, where DES serves as both a data collection mechanism (via barcode scanning) and a workflow structuring tool that maps these events into a conceptual digital twin for continuous tracking and visualization of care processes. Data-driven process simulation has been explored for capacity management applications in healthcare^24^, with a case study in a Belgian hospital’s radiology department demonstrating its utility in informing decisions on equipment requirements, waiting area capacity, and staffing needs.

Although patient flow simulations capture the movement of individuals through physical care environments, current studies often overlook the digital workflows that underpin clinical decision-making, such as the processing of medication orders, lab tests, and diagnostic imaging requests within the EHR system. This gap is particularly significant given the critical role that electronic order management plays in reducing delays and minimizing errors. Moreover, HIT-related challenges, such as poor system interoperability, human data entry errors, and unintended EHR system functionalities, further complicate electronic order processing. Issues such as system downtimes^25,26^, alert fatigue^25,27,28^, and inefficient clinical decision support mechanisms have been identified as persistent threats to patient safety and workflow efficiency in EHR environments^29,30^. This study takes the first step in modeling the flow of electronic orders within EHR systems.

To address the gap, this study proposes a DES framework tailored to reconstruct and evaluate electronic order workflows. First, the general simulation-driven order flow decision framework is introduced. The simulation models are then developed for the EHR order flows of five healthcare facilities based on exemplary data mapping (e.g., OASIS). The simulation is parameterized with empirical transition time distributions and validated against observed time-in-system metrics. Second, using one VHA healthcare facility as an example, the study describes how the impact of electronic order surges, resource capacity constraints, and transition probabilities can be evaluated and how order trajectory changes are visualized based on simulated results under different configurations. Finally, network analysis further reveals the relative centrality and importance of specific states and transition edges within the workflow. The approach provides a scalable data-driven foundation for evaluating EHR workflow performance under different settings, identifying trajectory variations, and supporting real-time anomaly detection and alerts.

## Results

Five VHA healthcare facilities were selected to evaluate the applicability of the proposed simulation framework (further details are shown in Fig.11). To standardize and interpret the heterogeneous order action logs generated by VHA’s EHR system, the data mapping protocol OASIS (Organization for the Advancement of Structured Information Standards) was adopted. OASIS is a nonprofit consortium that drives the development, convergence and adoption of open standards for the global information society^31^. It provides a generalized state-transition model widely used in business process management. OASIS-derived state mappings (further details are shown in Fig. 13) were applied on the raw EHR Consult order actions to improve the interpretability of the order logs, which standardizes the order processing states as *Created*, *Ready*, *Reserved*, *InProgress*, and *Completed* and the abnormal states as *Failed*, *Exited*, and *Error*. The simulation mirrors the state transitions and samples empirical transition durations from historical data. The following results include a model validation step followed by what-if scenario analyses on one facility from three aspects—electronic order surge, transition dynamics, and network analysis—to better understand the system dynamics and performance bottlenecks.

### Model validation

The transition probability for each order transition path in the simulation was estimated by using the relative frequencies of observed transitions in historical order flow data, and the transition duration was sampled directly from empirical distributions to capture variability without imposing parametric assumptions. Alternatively, the statistical distributions can be fit for transition durations.

**Fig. 1:**
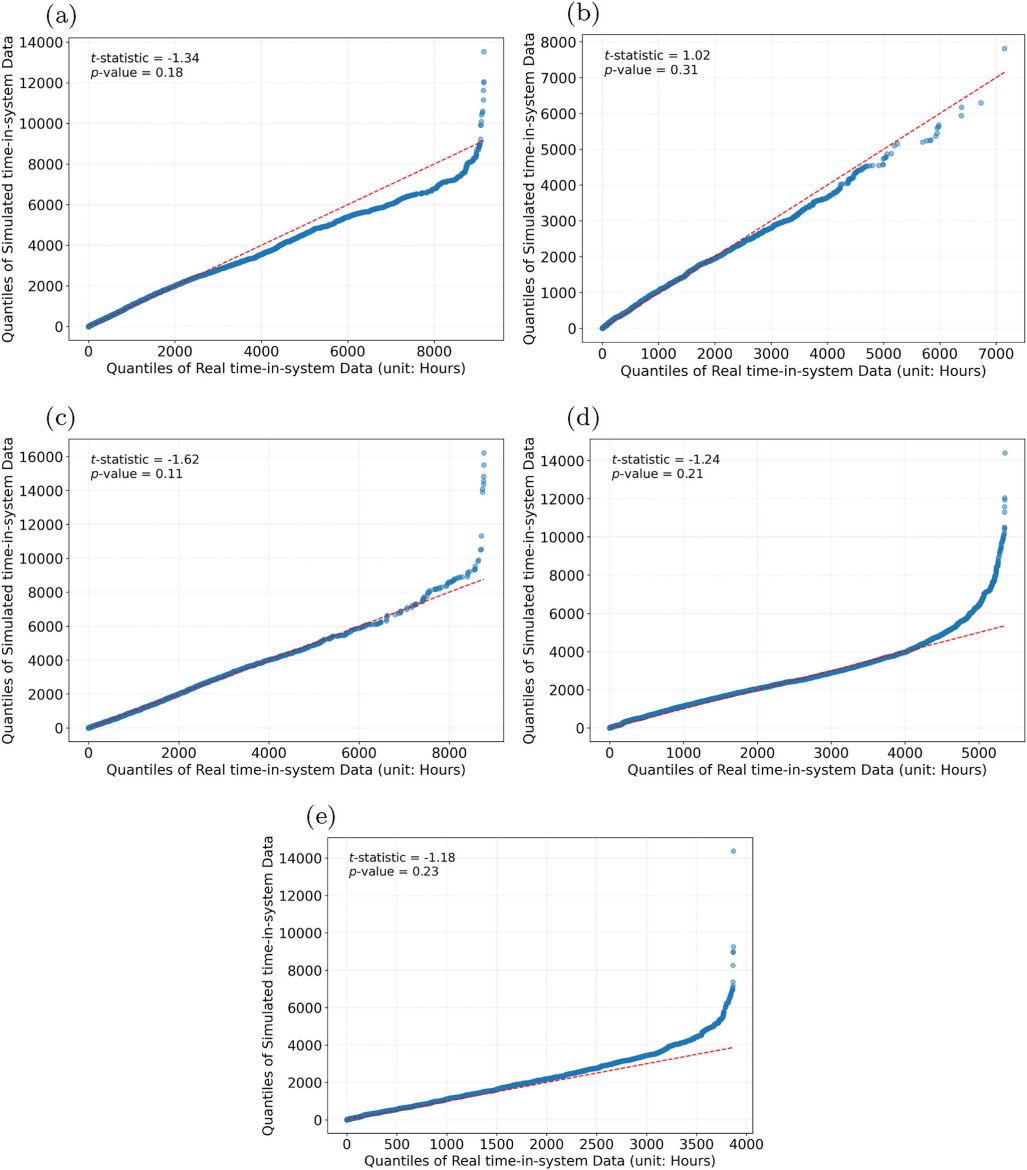
Comparison of observed and simulated time-in-system datasets across facilities. **a** Facility A. **b** Facility B. **c** Facility C. **d** Facility D. **e** Facility E. The Q-Q plots illustrate the correspondence between simulated and empirical datasets of time-in-system. Each point represents a corresponding quantile in the two datasets, and the red-dashed line indicates perfect concordance where the two datasets are identical.

Model performance was assessed by comparing simulated and observed time-in-system distributions, a key operational metric in electronic order processing. Time-in-system refers to the total elapsed time between when an order is first created and when it reaches its terminal state (e.g., *Completed* or abnormal states). This metric captures all intermediate waiting, processing, and decision steps associated with the handling of the order. Quantile-quantile (Q-Q) plots were used to visually assess concordance between the simulated and empirical distributions (Fig. 1). Across all five facilities, the Q-Q plots demonstrated strong agreement in the lower and central quantiles, and these results suggest that the models can accurately replicate typical processing patterns. Slight deviations in the upper quantiles were also noted, indicating that the simulations occasionally produced longer-than-observed process times. However, this is consistent with the use of some heavily tailed empirical samples. Given the potential operational impact of extreme values in healthcare (e.g., prolonged delays in diagnostic or therapeutic orders), we did not exclude outliers or truncate distributions during simulation in order to fully retain the risk landscape. This could enable the simulation to surface rare but critical scenarios which may warrant targeted analysis in other applications like exception handling.

To better statistically quantify the model accuracy, two sample *t*-tests were performed to compare the mean time-in-system between simulated and observed data. These tests yielded *t*-statistics ranging from -1.62 to 1.02 with p-values between 0.11 and 0.31 across all facilities. Thus, it is safe to conclude that no statistically significant differences were detected at the 95% confidence level.

**Fig. 2:**
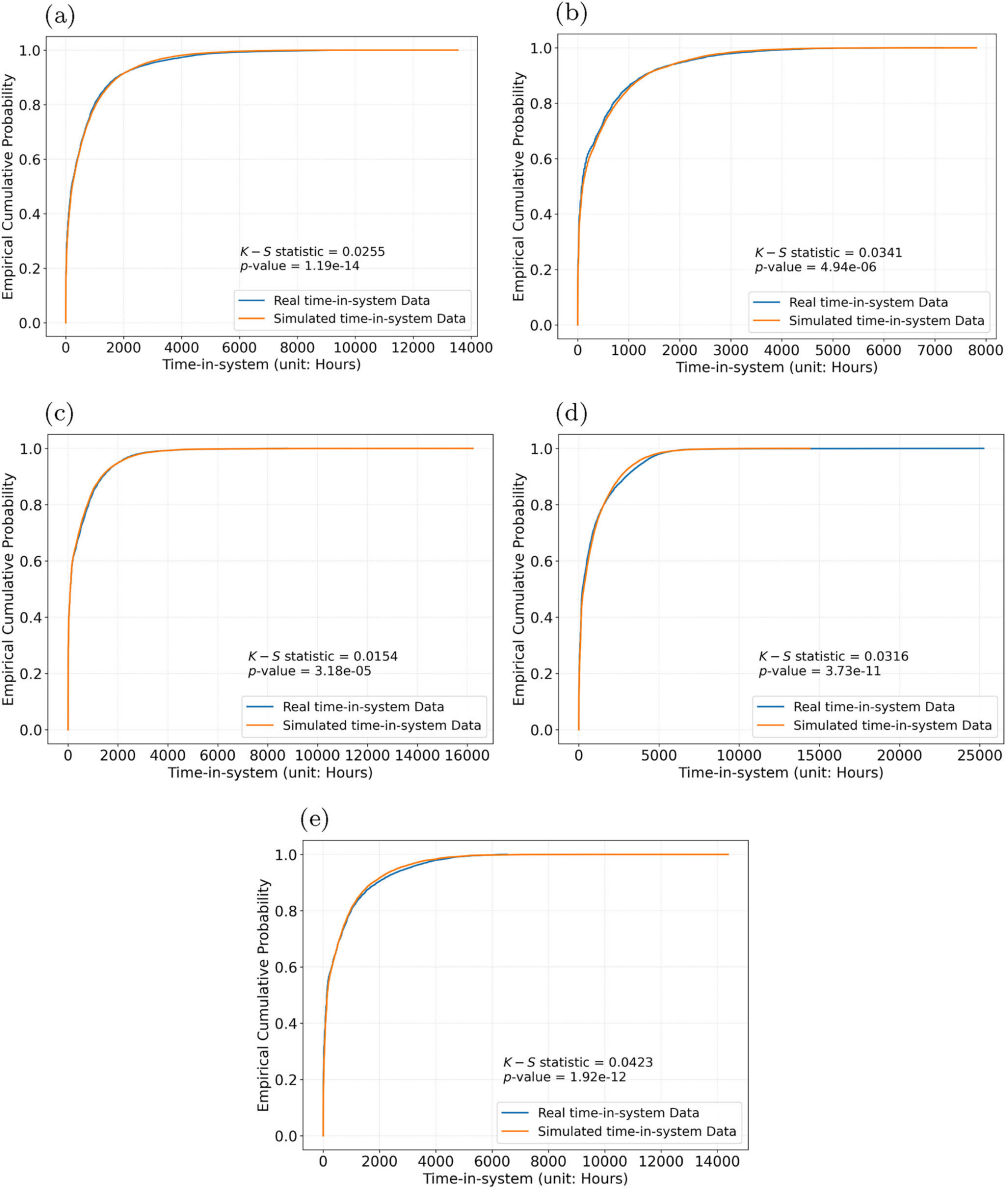
Empirical cumulative distribution functions (ECDF) of observed and simulated time-in-system datasets across facilities. **a** Facility A. **b** Facility B. **c** Facility C. **d** Facility D. **e** Facility E. The ECDF plots compare the distributional alignment between simulated and real time-in-system data. Each curve represents the cumulative probability of order duration for one dataset. The degree of vertical separation between the curves reflects the maximum difference in cumulative distributions, which is quantified by the K-S test statistic.

Supplemental to the *t*-tests for comparing mean performance, two-sample Kolmogorov-Smirnov (K-S) tests were also conducted to assess differences between the simulated and real time-in-system distribution. Empirical cumulative distribution functions for each facility are shown in Fig. 2, with the K-S statistics ranging from 0.0154 to 0.0423. These K-S statistics represent the maximum vertical distance between the two cumulative distributions and are uniformly small, which indicates close agreement in distributional shape. Although the corresponding *p*-values are extremely small, this is a known sensitivity issue of K-S test under large sample sizes^32,33^ and does not necessarily imply meaningful practical differences.

Additionally, We examined the Interquartile Range (IQR) for each facility to assess how well the simulation reproduces the spread of order durations. Table 1 summarizes the Q1, Q3, and IQR values for both real and simulated time-in-system data. Because the IQR captures the dispersion of the central 50% of the distribution, it provides a robust measure of system variability. Across all facilities, the IQR ratio fell within a narrow ± 10% range for the system level analysis, although no universal threshold exists for simulation validation.

**Table 1:** Interquartile Range (IQR) Comparison of Real and Simulated time-in-system (Hours) Statistics Across Facilities

Metric	Facility A	Facility B	Facility C	Facility D	Facility E
Sample Size	50,371	11,088	46,578	24,652	15,342
Q1 (Real Data)	23.27	15.66	3.67	48.42	19.59
Q1 (Simulated)	26.32	18.03	4.01	65.79	25.48
Q3 (Real Data)	813.66	533.72	642.69	1169.74	780.25
Q3 (Simulated)	836.22	581.79	580.61	1203.37	745.56
IQR (Real Data)	790.38	518.06	639.02	1121.31	760.67
IQR (Simulated)	809.90	563.76	576.60	1137.58	720.08
IQR ratio	1.02	1.09	0.90	1.01	0.95

Q1: 25th percentile, Q3: 75th percentile, IQR ratio = IQR(Simulated)/IQR(Real Data).

In summary, these results indicate that the models could reproduce both the structure and variability of real-world electronic order workflows and the simulation outputs are valid for downstream scenario analysis.

### Performance comparison

**Fig. 3:**
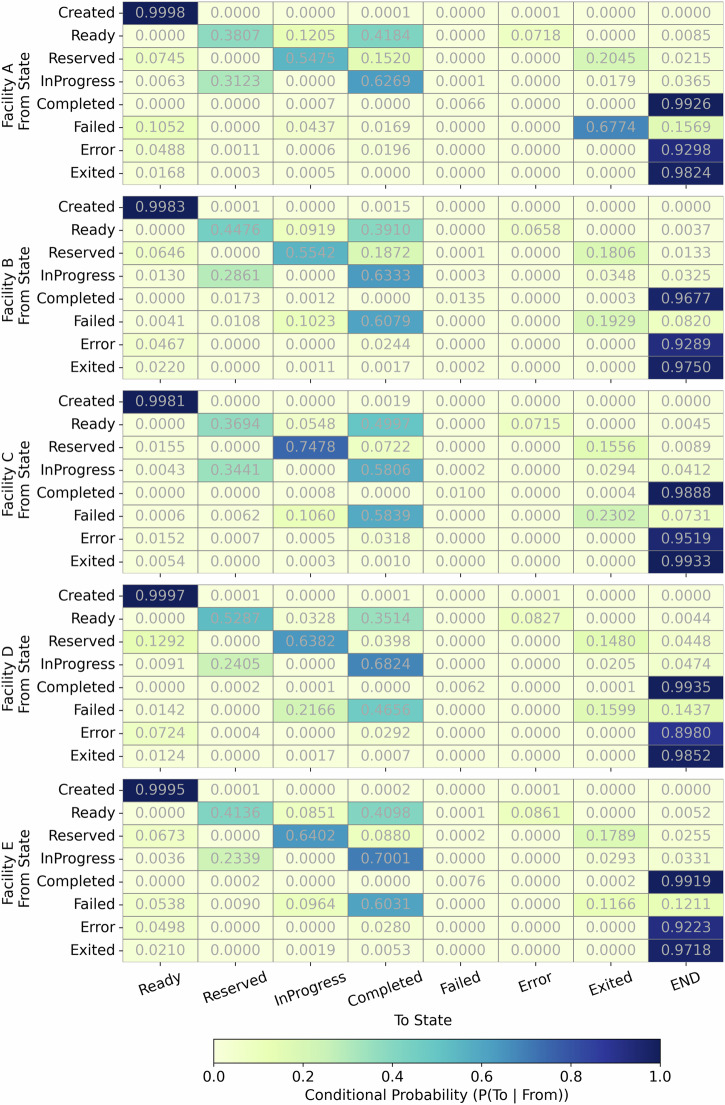
Row-wise conditional transition probabilities at workflow states. Each value is the transition probability from a given state (rows) to another possible state (columns) in the simulated order flows. Probabilities were computed as *P*(*B*∣*A*) = *Frequency*(*A* → *B*)/∑_*j*_Frequency(*A* → *j*) with rows normalized to 1. The *END* state means that order is terminated with the state.

**Fig. 4:**
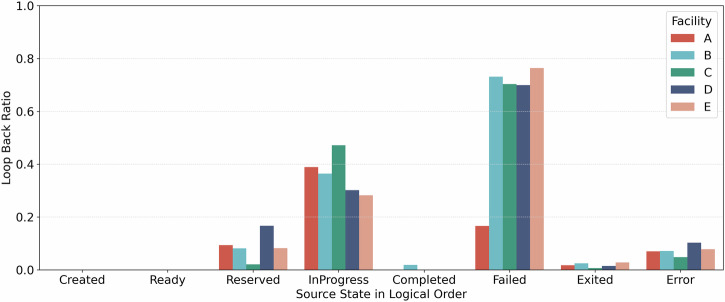
Loop-back ratios by workflow states across facilities. This loop-back ratio is calculated based on the defined logical workflow sequence: *Created* → *Ready* → *Reserved* → *InProgress* → *Completed* → *Failed* → *Exited* → *Error*. It measures the proportion of transitions returning to any earlier state in the defined sequence.

To further characterize order process behavior, conditional transition probabilities (Fig. 3) and order loop-back ratios at different workflow states (Fig. 4) were analyzed by using simulated data. Note that the empirical transition probabilities in real data can be directly computed from the frequency directly follows graph; for example, the probability values for Facility D can be derived from the flows shown in Fig. 15 for comparison. Although the overall workflow structure was generally consistent across facilities, as shown in Fig. 3, the transitions from *Created* to *Ready* occurred with near certainty, which reflects a potential standardized order initial processing procedure. However, operational variations emerged in transition dynamics and resolution patterns for orders in abnormal states. For example, the orders move from *Reserved* to *InProgress* with probabilities varying from 0.55 to 0.75 across facilities. The handling of orders in the *Failed* state notably differed: in Facility A, *Failed* orders led to *Exited* more often, whereas in other facilities, it was typically resolved to *Completed*, which suggests large variations in order-retry mechanisms or correction policies.

Based on the user-defined logical sequence of *Created* → *Ready* → *Reserved* → *InProgress* → *Completed* → *Failed* → *Exited* → *Error*, the loop-back ratio in Fig. 4 is constructed based on Fig. 3 by row-wise aggregating the transition probabilities to previous states. This ratio provides additional insight into order rework frequency. Additionally, for facilities B–E, the *Failed* state consistently exhibited the highest loop-back ratios (often > 0.7), indicating more frequent reentry into prior workflow stages (mostly to *InProgress* or *Completed*). This is consistent with order abnormal recovery or rework cycles. However, the loop-back ratio of *Failed* orders in Facility A is lower (< 0.2), and these orders have a high probability of flowing to *Exited* (Fig. 3). States such as *InProgress* also showed moderate loop-back patterns with ratios ranging from 0.3 to 0.45, which could indicate order reassessments or additional tasks. In contrast, terminal states such as *Completed* and *Exited* were rarely revisited, which reflects a proper end-state designation within the order life cycle.

These differences in process handling across facilities likely reflect the underlying heterogeneity in operational procedures, staff levels, and workload intensity. Moreover, identifying those states with high variability or recurrent loops may inform targeted interventions to reduce potential repetitive work and streamline transitions.

In the following three subsections, a series of simulation experiments was conducted to answer what-if questions such as *how will the system respond if the electronic order increases or the processing resources have been limited?* Facility D was selected as a representative example for the following scenario analyses. The simulation period was set to 2 years with a 1 h time resolution.

### Electronic order surge

Multiple order surge scenarios were simulated by increasing the order arrival rate and adjusting resource capacity constraints. The following scenarios were considered:

- *max_capacity_baseline:* This baseline scenario imposes no resource capacity limitations on any transition path. It allows all paths to concurrently process an unlimited number of orders. *max_capacity_arrival_+20%* represents the same configuration with a 20% increase in order arrival rate.
- *2000_capacity_baseline:* In this scenario, a resource capacity limitation of 2000 orders is imposed on the *Reserved* → *InProgress* and *InProgress* → *Completed* transitions, meaning that no more than 2000 orders can be processed simultaneously on these two paths. Any additional orders are held in the *InProgress* queue for *Reserved* → *InProgress* and the *Completed* queue for *InProgress* → *Completed*. *2000_capacity_arrival_+20%* represents this same configuration with a 20% increase in order arrival rate.
- *1000_capacity_baseline:* This scenario is similar to the previous scenario but uses a stricter resource capacity limitation of 1000 orders on the *Reserved* → *InProgress* and *InProgress* → *Completed* paths. *1000_capacity_arrival_+20%* represents the same configuration with a 20% increase in order arrival rate.

**Fig. 5:**
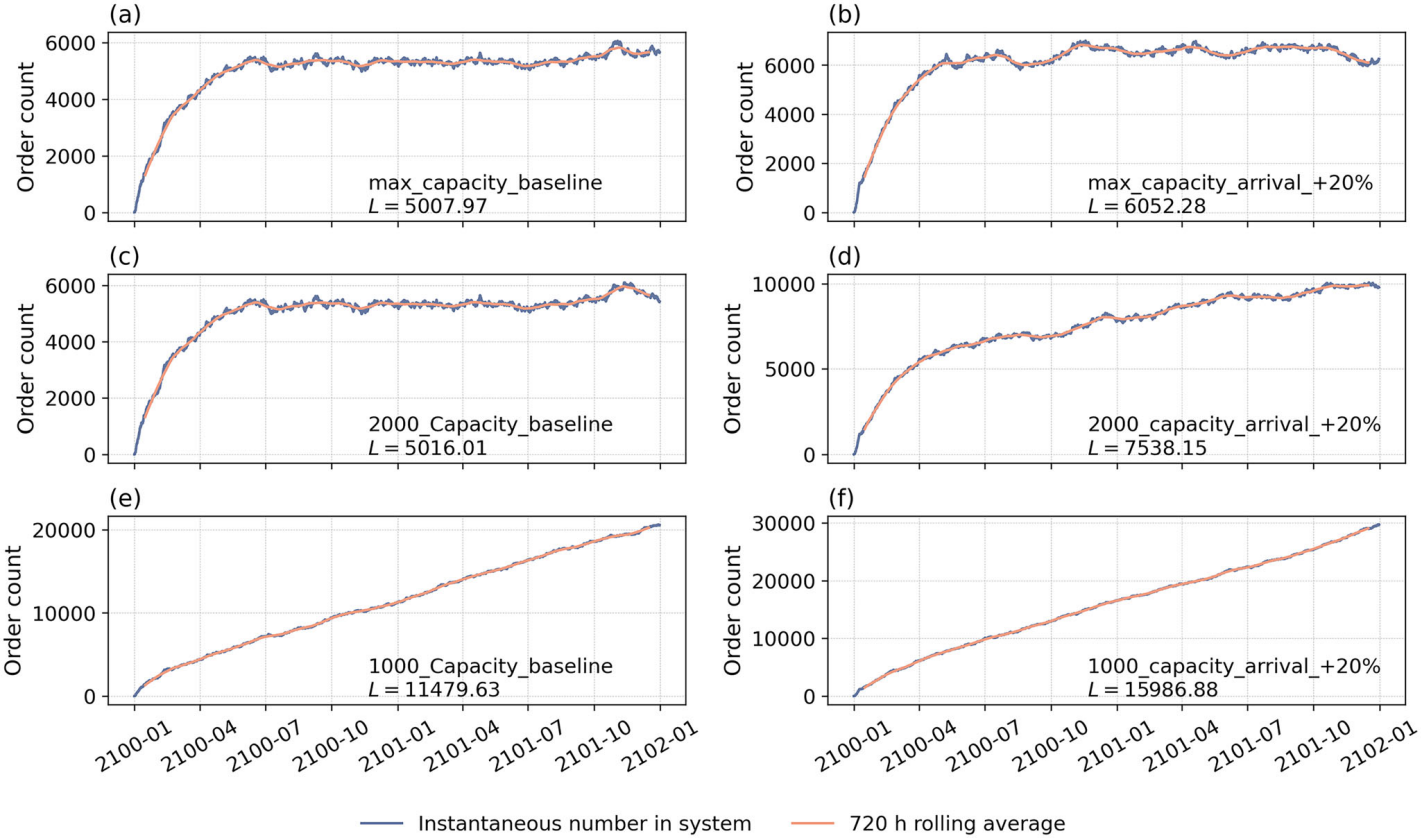
Instantaneous order number under different capacity and surge scenarios. **a** max_capacity_baseline. **b** max_capacity_arrival_+20%. **c** 2000_capacity_baseline. **d** 2000_capacity_arrival_+20%. **e** 1000_capacity_baseline. **f** 1000_capacity_arrival_+20%. This figure shows the order numbers in the system with a 1-month rolling average trend across six simulated scenarios. Resource capacity limitations are applied to the *Reserved* → *InProgress* (left) and *InProgress* → *Completed* (right) transitions with 2000 or 1000 order concurrent processing capacities.

Figure 5 shows the instantaneous order number in the system with a 1-month rolling average trend for each scenario’s simulation. In queuing theory, the average number-in-system, *L*, is also calculated from the simulated results. Theoretically, the average number-in-system in a stable system follows Little’s Law: *L* = *λ* ⋅ *W*, where *λ* is the average arrival rate, and *W* is the average time-in-system^34^. The results demonstrate that under baseline arrival rates, the order numbers in the system stabilize at a manageable level for max_capacity_baseline and 2000_capacity_baseline with similar values, whereas the 1000_capacity_baseline could not reach system stability within the simulated time range. As the arrival rate increases, max_capacity_arrival_+20% stabilizes at a slightly higher level than its baseline scenario. In contrast, the average number-in-system, *L*, starts to surge for 2000_capacity_arrival_+20%, with a 2522 increase (from 5016.01 to 7538.15), and it rises much more sharply under the 1000_capacity scenarios without stabilization.

The detailed system metrics for the various scenarios are summarized in Table 2. The state transition ratio is defined as (total occurrences of leaving the state) / (total order number). Notably, the transition ratio can be greater than 1 with the loop-back transition. The *Completed* transition ratio declines for the both capacity-limited scenarios when the arrival rate increases, which leads to a reduced throughput. The mean waiting time of the *InProgress* and the *Completed* queues suggests that although the overall completion remains dominant, the higher-order arrival rate increases the likelihood of delays and incomplete processing. Collectively, these results could help identify a system’s tipping point for demand- and resource-allocation planning.

**Table 2:** System performance metrics under different scenarios

Metric	max_capacity baseline	max_capacity arrival_+20%	2000_capacity baseline	2000_capacity arrival_+20%	1000_capacity baseline	1000_capacity arrival_+20%
Total Generated Order #	106,262	127,269	106,007	126,013	103,245	124,798
Ended Order #	100,586	121,023	100,573	116,225	82,689	95,093
*Ready* Trans. Ratio	109.44%	109.53%	109.57%	109.68%	109.32%	108.91%
*Reserved* Trans. Ratio	67.23%	67.40%	67.52%	67.75%	59.78%	52.90%
*InProgress* Trans. Ratio	45.95%	46.39%	46.11%	43.68%	30.34%	25.40%
*Completed* Trans. Ratio	74.84%	75.37%	74.97%	72.39%	60.75%	57.43%
*Failed* Trans. Ratio	0.45%	0.47%	0.44%	0.40%	0.36%	0.34%
*Exited* Trans. Ratio	11.49%	11.54%	11.59%	11.53%	11.06%	10.68%
*Error* Trans. Ratio	9.02%	9.04%	9.01%	9.12%	8.98%	8.88%
Avg. Number-in-System	5007.97	6052.28	5016.01	7538.15	11,479.63	15,986.88
InProgress Queue Mean (h)	0.00	0.00	0.00	0.00	2804.46	6420.90
Completed Queue Mean (h)	0.00	0.00	1.02	1714.91	4758.50	5340.15

Summary of simulation-derived metrics, including transition ratios, average number-in-system (*L*), and queue waiting times. Transition ratios are computed as the number of occurrences of leaving each state divided by total orders, and it can exceed 1 due to loop back transitions.

### Transition dynamics

Because the transition probabilities and processing times are the main input, the following simulations focus on examining how the order trajectory path will change if the probability or service time on certain paths is altered. A scenario with modified transition probabilities and capped service times is compared against the 2000_capacity_baseline configuration.

- *2000_capacity_prob_trans_change:* In this scenario, transition probabilities are dynamically adjusted partway through the simulation period. Specifically, at the end of the sixth month, the probability of transitioning directly from *Ready* to *Completed* was reduced from 0.354 to 0.104, and the probability of moving from *Ready* to *Reserved* was increased from 0.530 to 0.780. This adjustment represents a deliberate restriction of the shortcut path to completion. Additionally, to model the enforcement of a service level guarantee, the upper bound of processing time on the *InProgress* → *Completed* path is capped at 720 hours starting at the end of the first simulation year.

**Fig. 6:**
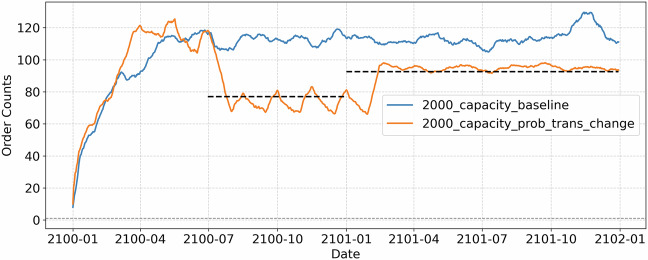
30-day rolling average of orders leaving the Completed state Two scenarios are simulated: 2000_capacity_baseline, in which transition probabilities and service times remained constant, and 2000_capacity_prob_trans_change, where the probability of transitioning from *Ready* directly to *Completed* is reduced to restrict shortcut to completion after the sixth simulation month. At the end of the first simulation year, the maximum processing time on the *InProgress* → *Completed* transition is capped at 720 hours to simulate a service level guarantee.

For the comparison, Fig. 6 shows the 30-day rolling average of orders leaving the *Completed* state over time. Under the 2000_capacity_baseline scenario, order throughput remains relatively stable following an initial ramp-up period. In contrast, the scenario with altered transition probabilities exhibits a marked decline in throughput shortly after the probability adjustments applied at the end of the sixth month. The throughput then stabilizes at a lower level before rising again after the 720 h service time cap was imposed on the *InProgress* → *Completed* path at the end of first simulation year. The turning points illustrate how dynamic changes in transition logic and processing can significantly impact system performance over time.

**Fig. 7:**
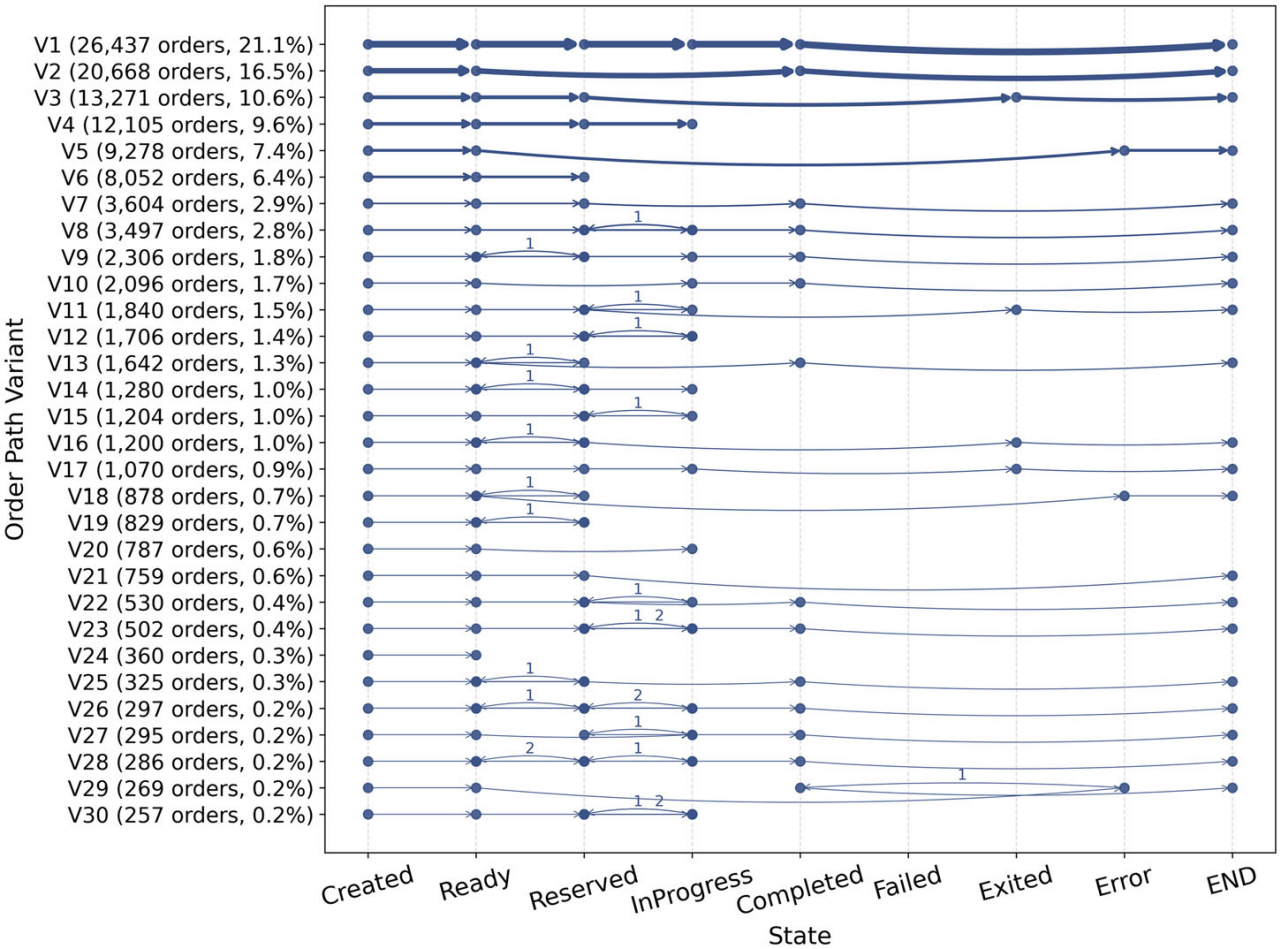
Top-30 order path variants (coverage: 93.7% orders) under the 2000_capacity_prob_trans_change scenario. Straight lines denote adjacent transitions, whereas curved arcs indicate non-adjacent (jumped) transitions. Numbers on loop-back arcs show the sequence of repeated loops if more than 1. For example, for path variant V23, 502 (0.4% of) orders follow the nine-step sequence with two recirculations through *InProgress → Reserved*. This is the path of V14 shown in Table 3. For path variant V28, the orders go through recirculation *InProgress* → *Reserved* and then *Reserved → Ready*. After modifying transition probabilities, the most common shortcut path (*Ready* → *Completed*) becomes less frequent and is replaced by longer paths with intermediate states.

To further understand the order path changes, the top-15 path variants for the 2000_capacity_baseline scenario are listed in Table 3, and the top-30 path variants for the 2000_capacity_prob_trans_change scenario are visualized in Fig. 7. Notably, after the transition probability change is applied in the 2000_capacity_prob_trans_change scenario, the most frequent order path in the baseline containing the shortcut *Ready* → *Completed* became the second-most frequent path. It is replaced by the path progressing through *Ready* → *Reserved* → *InProgress* → *Completed*, which reflects the intended shift in process flow dynamics.

**Table 3:** Top-15 order path variants in the 2000_capacity_baseline scenario

Variant	Orders	Path
01	37,046	*Created* → *Ready* → *Completed* → *END*
02	24,478	*Created* → *Ready* → *Reserved* → *InProgress* → *Completed* → *END*
03	8371	*Created* → *Ready* → *Reserved* → *Exited* → *END*
04	7900	*Created* → *Ready* → *Error* → *END*
05	3728	*Created* → *Ready* → *Reserved* → *InProgress* → *Reserved* → *InProgress* → *Completed* → *END*
06	2504	*Created* → *Ready* → *Reserved* → *Ready* → *Completed* → *END*
07	2323	*Created* → *Ready* → *InProgress* → *Completed* → *END*
08	2197	*Created* → *Ready* → *Reserved* → *Completed* → *END*
09	1870	*Created* → *Ready* → *Reserved*
10	1672	*Created* → *Ready* → *Reserved* → *InProgress*
11	1591	*Created* → *Ready* → *Reserved* → *Ready* → *Reserved* → *InProgress* → *Completed* → *END*
12	1292	*Created* → *Ready* → *Reserved* → *InProgress* → *Reserved* → *Exited* → *END*
13	744	*Created* → *Ready* → *Reserved* → *InProgress* → *Exited* → *END*
14	566	*Created* → *Ready* → *Reserved* → *InProgress* → *Reserved* → *InProgress* → *Reserved* → *InProgress* → *Completed* → *END*
15	547	*Created* → *Ready* → *Reserved* → *Ready* → *Reserved* → *Exited* → *END*

Each row shows a distinct sequence of workflow states (variant) and the count of orders following that path. Shorter paths (e.g., direct transitions from *Ready* to *Completed*) are more frequent under baseline probabilities. In the modified scenario, these shortcut paths are suppressed, and reliance on longer routes through *Reserved* and *InProgress* is increased.

**Fig. 8:**
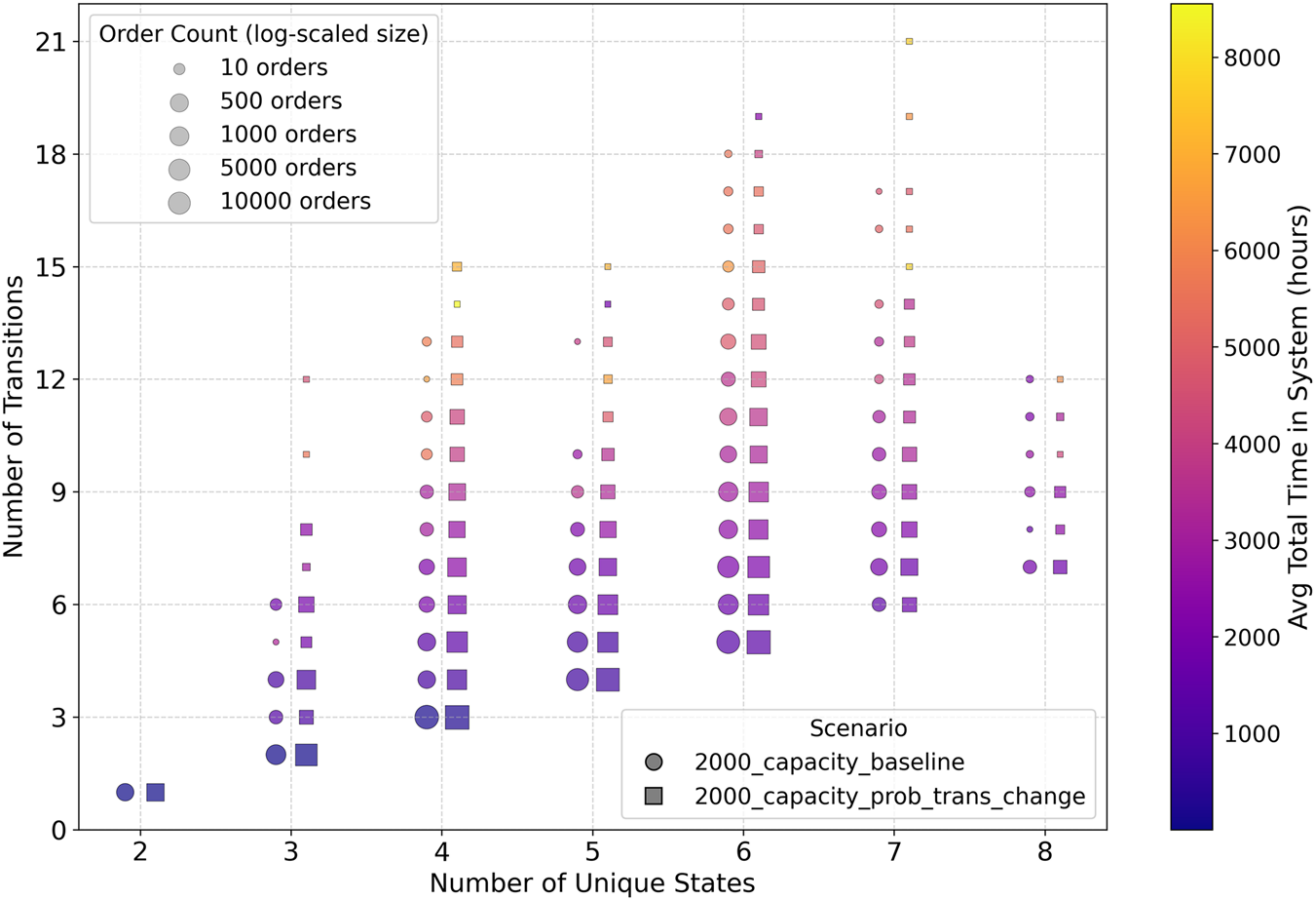
Distribution of unique states and total transitions per order path for the two scenarios. Each shape represents an order cluster sharing the same combination of total transitions and unique states. Its size is proportional to the log-scaled count of orders in the cluster. Color indicates the average time-in-system.

As an overview, Fig. 8 shows the distribution of the unique states versus the total number of transitions along the order paths. The scenario with modified transition probabilities exhibits longer and more complex transitions with a greater proportion of orders accumulating higher transition counts and extended durations. For example, some orders pass through as many as 21 transitions spanning 7 unique states. This demonstrates that modest changes in transition logic can produce substantial order recirculation with a high time-in-system, and simulation-based approaches can provide a valuable tool for assessing the effects of such routing adjustments or system glitches on process performance and throughput.

### Network analysis

**Fig. 9:**
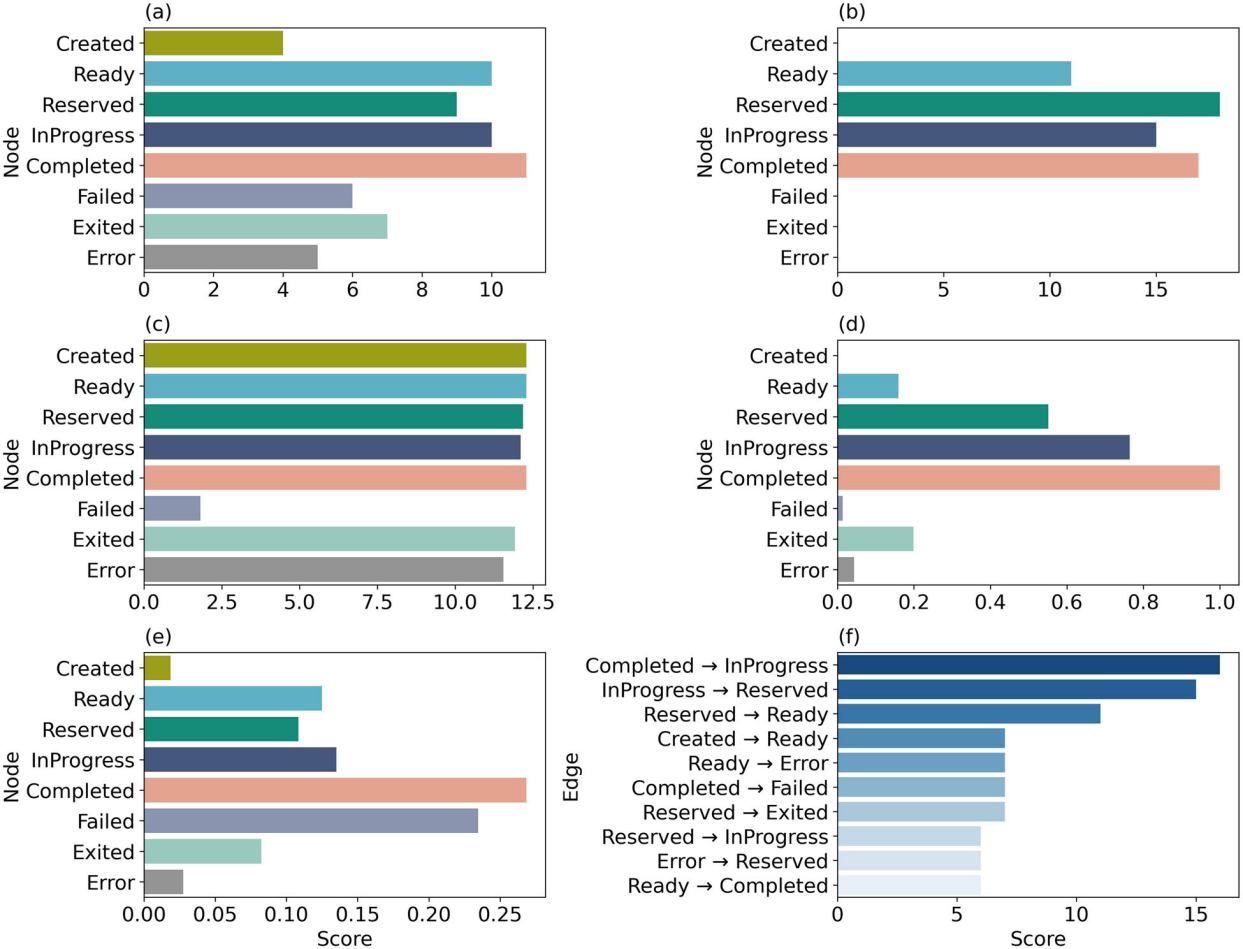
Summary of centrality metrics across nodes and edges. **a** Degree Centrality (Unweighted). **b** Betweenness Centrality (Weight: Duration/Count). **c** Closeness Centrality (Weight: Duration/Count). **d** Eigenvector Centrality (Weight: Count). **e** PageRank Centrality (Weight: Count). **f** Edge Betweenness Centrality. Degree, Betweenness, Closeness, Eigenvector, and PageRank centralities were computed to characterize each state’s structural importance. Formal definitions follow established formulations: Betweenness Centrality of node *v* as *C*_*B*_(*v*) = ∑_*s*≠*v*≠*t*_*σ*_*st*_(*v*)/*σ*_*st*_, where *σ*_*st*_ denotes the total number of shortest paths between nodes *s* and *t*, and *σ*_*st*_(*v*) counts how many of those paths pass through node *v*. In weighted graphs, the shortest paths are computed based on cumulative edge weights. The Betweenness Centrality of an edge follows the same principle. PageRank Centrality is computed as $PR(v)=\frac{1-d}{N}+d{\sum }_{u\in {\rm{In}}(v)}PR(u)/L(u)$, where *d* is a damping factor (commonly set to 0.85), *N* is the total number of nodes, In(*v*) represents the nodes linking to *v*, and *L*(*u*) is the out degree of node *u*.

In addition to the process simulation results, network-based analysis of a transition network was performed by using several centrality measures derived from the simulation results from the 2000_capacity_baseline scenario. This analysis quantifies the relative importance of each state and transition within the workflow graph and provides a structural perspective on how process dynamics emerge from the state transition topology. Multiple centrality metrics have been computed and summarized below^35,36^, and Fig. 9 shows the results:

- *Degree Centrality (unweighted)*: Counts the total number of direct incoming and outgoing edges attached to each node, regardless of transition frequencies or durations. Operational implication: Nodes with higher value is broadly connected and may act as a key interaction point. Stronger coordination may be required to manage diverse entry and exit pathways effectively.
- *Betweenness Centrality (weighted)*: Captures the extent to which a state lies on the shortest paths between all pairs of states. Higher betweenness implies greater control over information or order flows, which may define the state as a bridge or bottleneck. Operational implication: Higher betweenness implies greater control over information or order flows, which may define the state as a bridge or bottleneck. Delays here can cascade downstream, amplifying system wide congestion. Improve operational efficiency in such nodes can reduce systematic latency.
- *Closeness Centrality (weighted)*: Quantifies how close a node is, on average, to all other nodes in the network based on the sum of the shortest path lengths. Nodes with high closeness can quickly reach (or be reached by) all others. Operational implication: Nodes with higher value are centrally located in the network structure. It would be ideal for implementing real time monitoring or priority triage mechanisms.
- *Eigenvector Centrality (weighted)*: Measures not only the number of connections but also their quality by assigning higher scores to nodes linked to other high scoring nodes. This metric highlights influential hubs embedded in well-connected neighborhoods. Operational implication: Nodes with higher score reflect strategic influence within the work flow where they may benefit from enhanced audit logging or targeted quality assurance.
- *PageRank Centrality (weighted)*: Estimates the relative importance of nodes based on the idea that connections from important states contribute more weight, which tends to capture recursive influence within the process flow. Operational implication: High PageRank score suggest area where small issues could have outsized effects on other important nodes. These nodes may warrant periodic risk assessment.

**Fig. 10:**
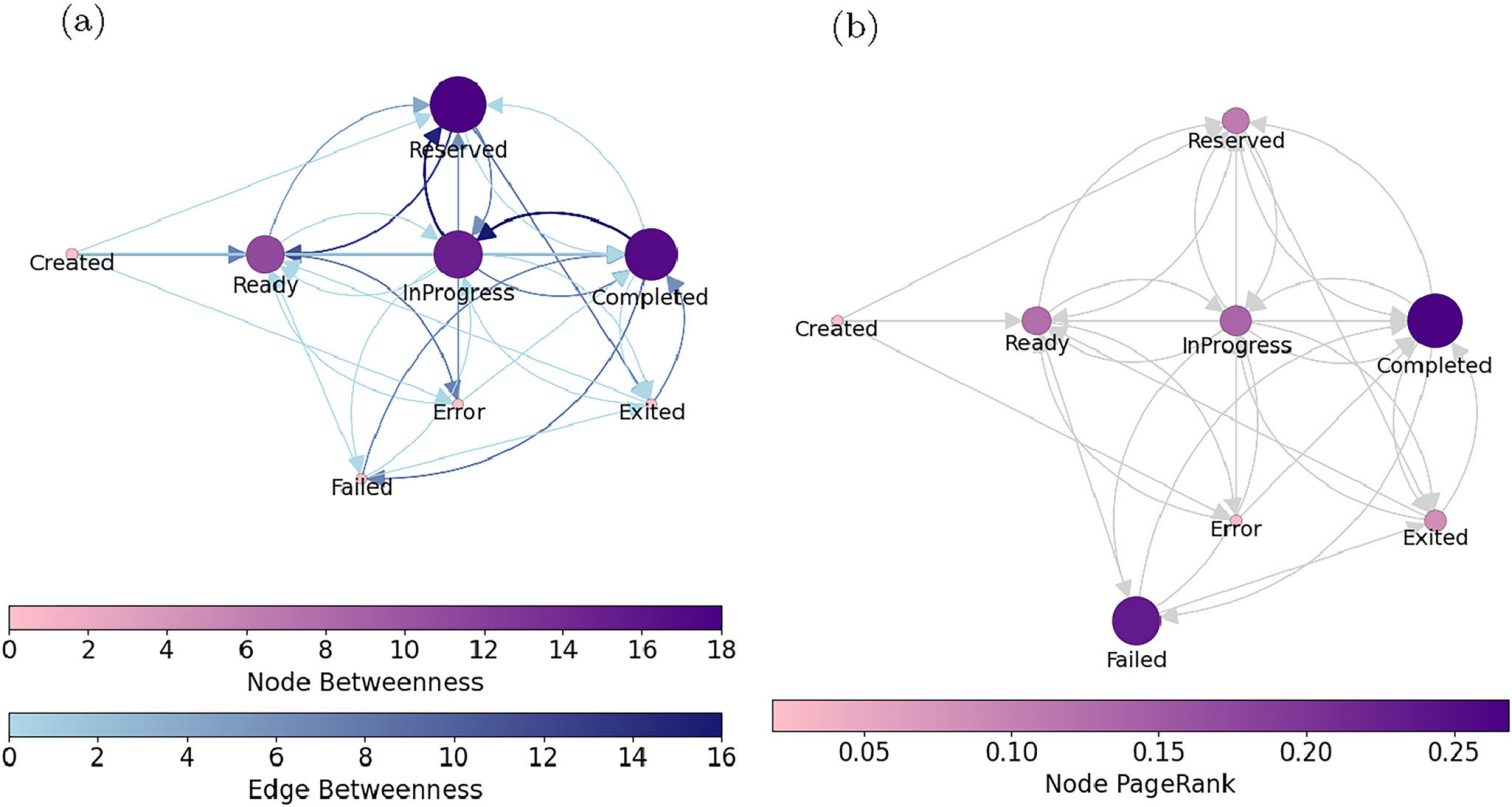
Betweenness and PageRank centrality graphs for the 2000_capacity_baseline scenario. **a** Betweenness Centrality (Weight: Duration/Count)–Node and Edge. **b** PageRank Centrality (Weight: Count). Each node represents a workflow state, and the edge thickness reflects connection strength. Betweenness centrality quantifies how frequently a state lies on the shortest weighted paths between all pairs of states. PageRank centrality captures recursive influence based on the importance of connected nodes.

For shortest path based measures (Betweenness and Closeness centralities), weights on the edges are defined as the average transition duration divided by the order count. This simultaneously accounts for time performance and order transition frequency. For Eigenvector and PageRank centralities, order count is used as the edge weight to emphasize the strength of connections. Among all nodes, *Reserved*, *Completed*, and *InProgress* continuously emerge as the most structurally significant states within the workflow. Specifically, *Reserved* exhibited the highest score in Betweenness Centrality, which indicates that it lies on many of the shortest weighted paths and functions as a critical bridge between other stages. *Completed* consistently dominated in PageRank and Eigenvector centralities, which indicates its strong connectivity and importance as the most frequent termination point. *InProgress* also showed overall high scores across centrality metrics, thereby echoing that it serves as a major processing hub. In contrast, the abnormal states *Failed*, *Exited*, and *Error* maintain lower centrality scores. The *Failed* state has a higher score in PageRank Centrality because it is connected to the most influential state node, *Completed*, and commonly appears as its subsequent state. The centrality graph for Betweenness and PageRank are shown in Fig. 10.

At the edge level, Betweenness Centrality further clarifies which transitions serve as critical conduits. The transition *Completed → InProgress* has the highest edge betweenness, followed by *InProgress → Reserved* and *Reserved → Ready*, which marks them as key structural links that facilitate recirculation in the network. In summary, these results illustrate and support the logic that *Completed* anchors most workflows, *Reserved* plays an outsized role in routing and rework, and *InProgress* serves as a major transit state. Together, they form the backbone of the order transition network.

## Discussion

This study developed and evaluated a DES framework to model EHR order flows. By reconstructing state transitions from real-world EHR data, the simulation replicated observed operational patterns, including time-in-system distributions and site-specific variability across facilities. Scenario analyses demonstrated that increased orders and limited resource capacity led to pronounced system congestion. Dynamic adjustments in transition probabilities and processing times further revealed that even modest changes in workflow behavior can drive substantial recirculation and process delays. Network-based centrality analysis has identified *Reserved*, *InProgress*, and *Completed* as structurally important states, which act as key hubs and potential bottlenecks within the order workflow. All the findings highlight the effectiveness and validity of simulation-based approaches in elucidating the interplay among routing logic, capacity constraints, and overall system performance.

This work provides several actionable insights for healthcare operations leaders and HIT professionals, directly grounded in the simulation and what-if scenario results:

- *Use simulation for safe policy testing and workflow design*: The demonstrated what-if scenarios show that the simulation framework can serve as a virtual controllable testing environment for evaluating proposed changes to workflow, policy, or resource allocation, which enables informed decisions prior to real-world implementation.
- *Plan for scalable surge response*: Simulation results under order surge conditions highlight the importance of contingency strategies such as adaptive prioritization protocols, staffing flexibility and elastic processing capacity are critical to sustain desired performance under stress.
- *Monitor critical states to detect emerging congestion*: Centrality analysis identified critical states such as *Reserved*, *InProgress*, and *Completed*. Monitoring metrics like active order volume or dwell time in these states (e.g., using rolling average) can enable earlier detection of congestion and support timely intervention.
- *Prevent rework cycles by identifying looping transitions*: In transition dynamics analysis, looping behaviors and extended process durations emerged. Automatic flagging excessive recirculation or order re-entries can help detect inefficiencies and trigger corrective workflows or alerts.
- *Leverage transition dynamics as early warning indicators*: Shifts in transition duration or path probabilities can serve as leading indicators of workflow drift. Tracking these metrics over time supports proactive operational adjustments to scheduling, workload distribution, or policy enforcement.

Several limitations should be acknowledged and explored in future work. In this study, the order states were aggregated at a high level, which potentially obscures finer-grained transitions that could further clarify more process dynamics. The simulation outputs, including delays, rework loops, and order path variants also provide clinically interpretable signals that can guide workflow optimization in practice. Although the developed framework does not model detailed task level clinical activities, it serves as a system level diagnostic tool that highlights where operational bottlenecks arise and where more detailed clinical investigation may be warranted. The detailed process within each state should be investigated more thoroughly to enable effective root cause analysis. Transition probabilities and durations were assumed to remain stationary over time, which may not fully capture evolving practice patterns or system updates. Additionally, factors such as user behavior, organizational culture, and policy constraints were beyond the scope of this analysis. For future studies, the simulation can be expanded to model a more comprehensive healthcare network by incorporating additional healthcare providers and simulating referral pathways. Another promising direction is to integrate online predictive models with adaptive simulation to support real-time monitoring and decision support.

Overall, this study demonstrates that DES offers a robust quantitative framework to analyze EHR order processing performance, identify structural bottlenecks, and evaluate the impact of operational changes before deployment. By enabling data-driven scenario analysis and highlighting actionable insights, this approach supports evidence-based decision-making to enhance the efficiency of electronic order workflows in complex healthcare environments.

While the current framework is implemented using data from VHA EHR system, its core components are designed to be broadly adaptable to other healthcare settings. The DES engine and network based analysis pipeline are data-driven and modular, which can be transferable to systems given timestamped order level event logs. Key elements that can be generalizable include the structure of the simulation model and scenarios design, order path variation discovery and the network centrality analysis methods. However, two main areas need further adaptation: 1) the mapping of raw order events. The used OASIS-derived states mapping needs to be reconsidered since order process states may vary by institutions or HIT vendors; 2) transition probabilities and durations may needs different calibration approach based on local data characteristics and practice guidelines. With these customization, the framework can support EHR order workflow evaluation in a variety of healthcare environments.

## Methods

### EHR order flow simulation framework

Fully representing EHR systems would require modeling numerous detailed clinical tasks, workflows, staff assignment and operational constraints. The intention here is to develop a framework that can provides system level abstraction that first localizes where delays, rework loops or anomalies occur which serves as actionable entry points for investigators to conduct more detailed root cause analysis.

**Fig. 11:**
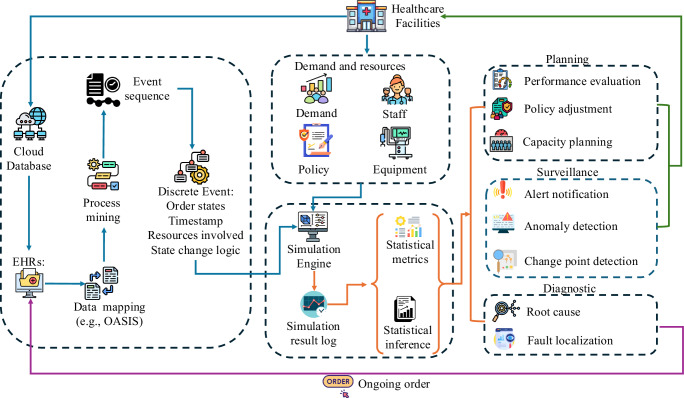
The discrete event–based EHR order flow simulation framework outlines the basic decision flow for applying simulation-driven decision support. Historical EHR-derived event sequences serve as the primary input to construct the order process flow. Demand and resources data provide additional configuration settings. After calibration and validation, the simulation output can be used to support decision-making in planning, surveillance, and diagnostic applications.

In the proposed discrete event–based EHR order flow simulation framework (Fig. 11), patient order information is stored in data warehouses. These EHR order records are preprocessed through denoising, mapping, and segmentation steps. Key process patterns are then extracted by using process mining to construct the order event handling workflows. Demand prediction and resources required at each workflow state are also incorporated into the simulation as configuration settings. Institutional policies serve as the underlying logic that guides order progression in the network.

After the simulation model is calibrated and validated with domain experts, statistical indicators derived from simulation results can be used to evaluate policies and plan related resources. In rolling-window simulations, deviations in key metrics can be used as alerts for potential change points or anomalies in the system. Additionally, when abnormal delays or deviations are detected in the order pathways, the simulation can be easily rerun under counterfactual conditions to isolate the components or processes that are most likely responsible for the disruption. Compared with raw EHR-order, data-based retrospective detection methods, this simulation capability enhances and encourages proactively identifying and mitigating systemic inefficiencies and failures.

With the integration of real-time resource data streams and predictive capabilities, the framework has the potential to evolve into a healthcare digital twin, which could continuously mirror the operational state of the system to support real-time decision-making in an operational environment.

### Raw data mapping of EHR order

**Fig. 12:**
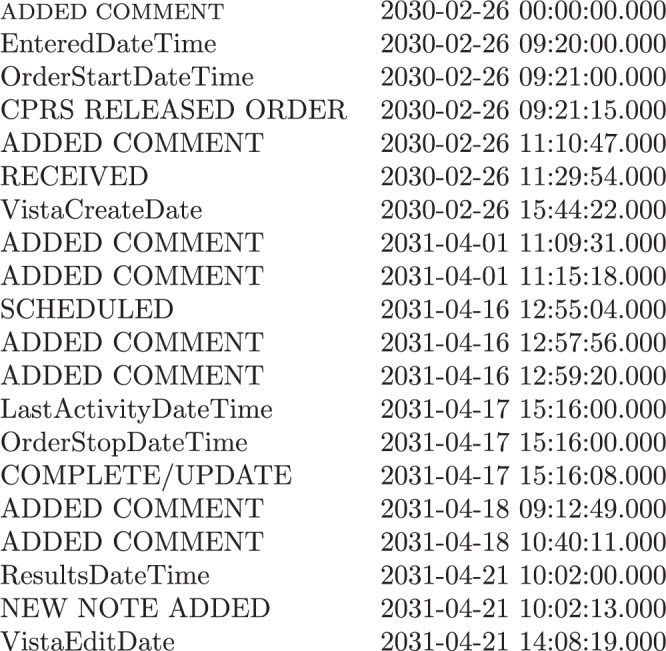
Sample Consult order event sequence.

To simulate the electronic order flow, discrete events of the clinical orders must be accurately and uniformly reconstructed. Taking the VA’s CDW as an example, it contains extensive clinical data, yet the event sequences for patients or computerized provider order entries are not readily available. The primary challenges in extracting meaningful sequences stem from the relevant information typically being dispersed across disparate database tables. This information can sometimes only be discerned through examination and denormalization of the data, making it an arduous and laborious task to effectively consolidate and interpret event sequences. After identifying all timestamp records that are assumed to represent a transaction on an order, all tables that have some form of activity or status update associated with that order are recorded, and then the raw data is obtained as a time-sorted event sequence ^37^. This data mining process can be applied to the orders in different domains (e.g., Consults, Radiology, Labs, outpatient prescriptions) to obtain the order event sequence ^38^. For example, a sample Consult order event sequence is shown in Fig. 12. The real date and time are masked for privacy protection.

**Fig. 13:**
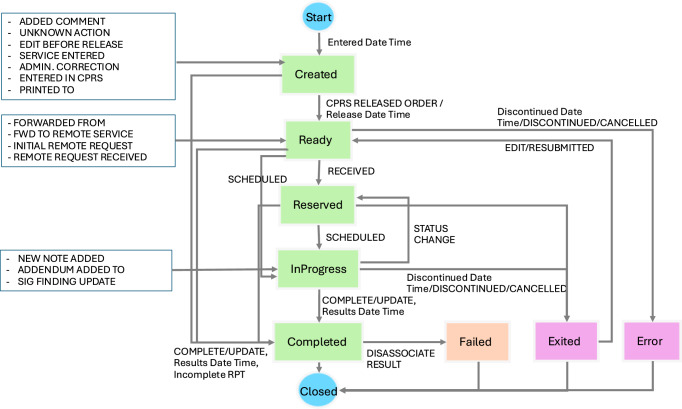
Mapped OASIS human task state transition diagram. The diagram illustrates the standardized life cycle of an order, including common paths from *Created* through *Completed* as well as less frequent transitions to *Failed*, *Exited*, or *Error*. States and transitions were defined in collaboration with subject matter experts to reflect operational realities in the VHA EHR system.

To enhance the interpretability of event logs, this study adopted the state transition diagram from the OASIS Web Services human task standard. This approach maps various process-related activities throughout their life cycles into OASIS human task state transitions. In collaboration with subject matter experts working with the VistA EHR system and the VHA CDW, this study established mapping rules for Consult orders (summarized in Table 4). Specific DateTime columns and consult activities are aligned with corresponding OASIS states, as depicted in Fig. 13.

**Table 4:** Consults dataset mapping to OASIS human task states^38^

OASIS Human Task State	Consult Date Columns	Consult Activities and Other Events
CREATED	EarliestDate, EnteredDateTime	
READY	FileEntryDatetime, OrderStartDateTime, ActivityDateTime, ReleaseDateTime	NW
RESERVED	SignedDateTime, RequestDateTime, VistaCreateDate, [PatientAppointmentDateTime]	Received, CPRS released order
INPROGRESS	ActivityDateTime, ChartCopyPrintedDateTime, OccurrenceDateTime, LapsedDateTime, VistaEditDate, OrderActionDateTime, NurseVerifiedDateTime, ClerkVerifiedDateTime, ChartReviewedDateTime, DiscontinuedHoldUntilDateTime, OrderActionFlagDateTime, OrderActionUnflaggedDateTime, InterventionDate, PharmacistCommentsDateTime, ActivityEntryDateTime, RemoteFilingDateTime	Scheduled, printed to, added comment, incomplete rpt, new note added, forwarded from, status change, sig finding update, edit/resubmitted, addendum added to, disassociated result, FWD to remote service, admin. correction, dc, missing
COMPLETED	LastActivityDateTime, OrderStopDateTime, CompletedDateTime, ResultsDateTime	Complete/update
SUSPENDED	–	Waiting states with time interval
FAILED	Completed with DiscontinuedDateTime recorded	Disassociate result
ERROR	DiscontinuedDateTime	Discontinued
EXITED	DiscontinuedDateTime (else: not at Completed or Ready)	Discontinued, canceled with package number
CLOSED	VistaEditDate	Canceled without package number

This table summarizes how specific VHA CDW timestamp columns and activity labels were aligned to canonical OASIS states to standardize event representation. For example, fields such as *EnteredDateTime*, *OrderStartDateTime*, and *VistaCreateDate* were mapped to *Created*, *Ready*, or *Reserved* states, whereas discontinuation events were categorized as *Error*, *Exited*, or *Closed*. The activity value “NW” means new consult. This mapping simplifies downstream simulation and enables consistent detection of process variation.

This mapped state transition diagram effectively reduces dataset complexity and provides actionable insights. Domain experts can utilize the diagram to identify irregular and rare transitions by analyzing transition path statistics. The dataset analysis reveals that most orders follow a standard transition pattern: *Created*, *Ready*, *Reserved*, *InProgress*, and *Completed*. However, irregular and infrequent transitions to states such as *Failed*, *Exited*, and *Error* may indicate potential issues. A sampled order case from the VHA CDW that concluded with a *Failed* state is presented in Table 5.

**Table 5:** Example Consult order ending in a Failed state

Activities	Datetime
EnteredDateTime	2021-12-23 15:33:00.000
OrderStartDateTime	2021-12-23 15:33:00.000
CPRS RELEASED ORDER	2021-12-23 15:33:45.000
RECEIVED	2021-12-23 15:50:13.000
VistaCreateDate	2021-12-23 21:02:32.000
SCHEDULED	2022-01-10 11:48:51.000
STATUS CHANGE	2022-03-30 15:06:38.000
INCOMPLETE RPT	2022-03-31 14:29:46.000
INCOMPLETE RPT	2022-03-31 15:17:06.000
ResultsDateTime	2022-04-03 14:21:00.000
OrderStopDateTime	2022-04-03 14:21:00.000
COMPLETE/UPDATE	2022-04-03 14:21:34.000
DISASSOCIATE RESULT	2022-04-03 14:21:58.000
LastActivityDateTime	2022-04-03 14:22:00.000
VistaEditDate	2022-04-03 18:22:07.000

This sample record illustrates a representative Consult order that is concluded in a *Failed* state. The presence of activities such as *INCOMPLETE RPT* and *DISASSOCIATE RESULT* implies that this order did not complete successfully.

A more detailed explanation of the data extraction and mapping process can be found in previous studies ^37–40^. This study focuses specifically on EHR orders within the Consults domain.

### Simulation setup

**Fig. 14:**
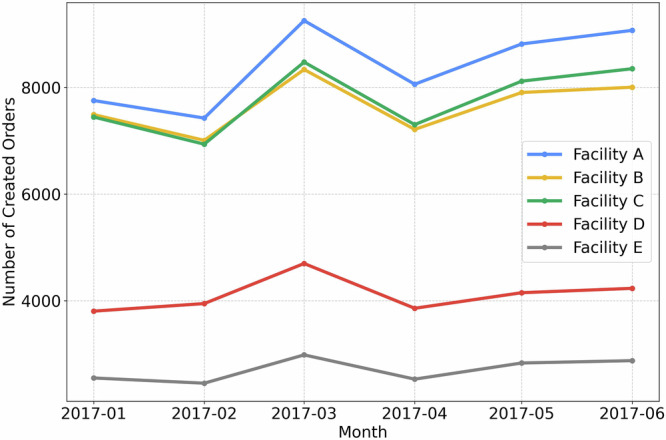
Monthly volume of created Consult orders in selected facilities. The Clinical Complexity Index classifies healthcare facilities as a range of Level 1a (highest complexity) to Level 3 (lowest complexity) based on patient volume and risk, scope of clinical services, and extent of research and teaching activities^42^. The facility classifications represented above include Level 1a (Facility A, 50,398 orders), Level 1b (Facility B, 45,972 orders), Level 1c (Facility C, 46,634 orders), Level 2 (Facility D, 24,694 orders), and Level 3 (Facility E, 16,221 orders). The observation period spanned from January to June of 2017. Higher-complexity facilities generally processed larger volumes of Consult orders each month.

By mirroring the mapped OASIS state transition diagram, DES is designed to simulate the order flow of selected facilities and assess their system performance. This approach enables a dynamic evaluation of process efficiency to enable the identification of bottlenecks and irregular transition patterns within the Consults workflow. Based on the availability of order data in the Consults domain, facilities at different complexity levels were selected. The selected date range spans from January 2017 to June 2017, and the monthly distribution of created orders is shown in Fig. 14.

**Fig. 15:**
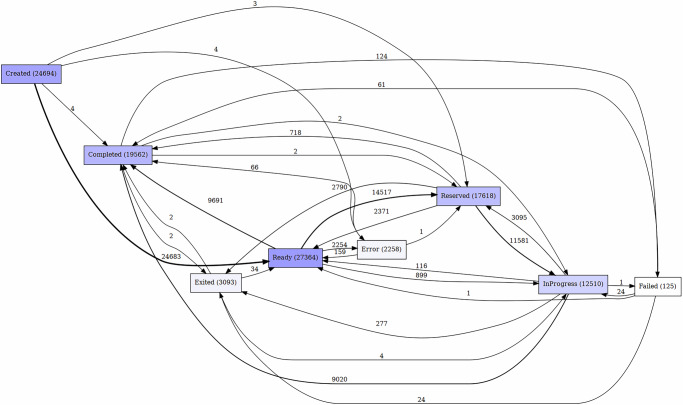
The frequency–directly follows graph of Facility D. Each node represents an order state, and the number inside the node is the total number of orders that entered that state during the observation period. Directed edges between nodes represent observed transitions between states, with the order number labeled on that edge. Edge thickness is proportional to transition frequency to emphasize high volume transitions. This frequency-based visualization aids in identifying dominant pathways and rare transitions within the Consult workflow for a selected facility. Time performance (e.g., average transition time) can also be added to the edge.

For a clearer illustration of the state transitions in the OASIS mapping of raw data, Facility D’s frequency–directly follows graph is shown in Fig. 15. This graph was generated by using the PM4PY process mining package^41^. For example, the raw order data in Facility D shows that, among the total 24,694 orders, 24,683 orders transitioned from the *Created* state to the *Ready* state with a mean transition time of 1 h. Additionally, 4 orders transitioned directly to the *Completed* state with an average duration of 3 months, and 4 orders transitioned to the *Error* state with an average duration of 5 months. Notably, the total number of outgoing transitions from a given state may be lower than the total number of orders in that state because some orders may still be active during data capture.

## Data Availability

The EHR order data used in this study was derived from VHA systems and is not publicly available owing to patient privacy and data use restrictions. Access may be granted upon request and approval by the VA.

## Code availability

The simulation framework and analysis scripts developed for this study are available from the corresponding author upon reasonable request.
